# Supplementary material for: Incidence of dry eye symptoms and behavioural-cultural risk factors among university students population in Jordan
Source: PLoS One. 2025 Aug 7;20(8):e0328235. doi: 10.1371/journal.pone.0328235 (PMC12331033; doi:10.1371/journal.pone.0328235)
Supplement: S1 File — (DOCX) [file pone.0328235.s001.docx]

**Questionnaire**

**Part One: Sociodemographic**

1. Gender: Male □ Female □
2. Age: __________________
3. Emirates: Dubai □ Abu Dhabi □ Sharjah □ Ajman □ Fujairah □ Ras Al-Khaimah □ Umm Al Quwain □
4. Hours of screen use (phone/computer) per day: _______________
5. Hours of AC conditioning per day: __________________
6. Use of contact lens: Never □ Sometimes □ Always □

**Part Two: OSDI**

1. Have you experienced sensitive eyes to light during the last week?

- Never
- Some time
- Half of the time
- Most of the time
- All the time

1. Have you experienced gritty eyes during the last week?

- Never
- Some time
- Half of the time
- Most of the time
- All the time

1. Have you experienced painful or sore eyes during the last week?

- Never
- Some time
- Half of the time
- Most of the time
- All the time

1. Have you experienced blurred vision during the last week?

- Never
- Some time
- Half of the time
- Most of the time
- All the time

1. Have you experienced poor vision during the last week?

- Never
- Some time
- Half of the time
- Most of the time
- All the time

1. Have problems with your eyes limited you in reading during the last week?

- Never
- Some time
- Half of the time
- Most of the time
- All the time
- Not applicable

1. Have problems with your eyes limited you in night driving during the last week?

- Never
- Some time
- Half of the time
- Most of the time
- All the time
- Not applicable

1. Have problems with your eyes limited you in working with a computer or bank machine (ATM) during the last week?

- Never
- Some time
- Half of the time
- Most of the time
- All the time
- Not applicable

1. Have problems with your eyes limited you in watching TV during the last week?

- Never
- Some time
- Half of the time
- Most of the time
- All the time
- Not applicable

1. Have your eyes felt uncomfortable in windy conditions during the last week?

- Never
- Some time
- Half of the time
- Most of the time
- All the time
- Not applicable

1. Have your eyes felt uncomfortable in low humidity areas (very dry) during the last week?

- Never
- Some time
- Half of the time
- Most of the time
- All the time
- Not Applicable

1. Have your eyes felt uncomfortable in air-conditioned areas during the last week?

- Never
- Some time
- Half of the time
- Most of the time
- All the time
- Not applicable

**Part Two: Smoking**

1. What type of tobacco do you mostly smoke?

- Regular Cigarette
- Electronic cigarette (Vape)
- Dokha (Medwakh  Pipe)
- Shisha (Mouassal)
- Shisha (Ajami)
- Non smoker

1. How often do you smoke tobacco?

- Daily smoking (at least 5 days per week)
- Weekly smoking (1-2 days per week)
- Monthly smoking (1-2 days per month)
- Non smoker

1. Where often do you smoke tobacco?

- Indoor areas
- Outdoor areas
- Non smoker

1. For how many years have you smoked tobacco? (Answer by zero if you are a non-smoker) (open-ended question)
2. On average, how many cigarettes (regular and/or electronic cigarettes) do you smoke per week? (Answer by zero if you are a non-smoker) (open-ended question)
3. On average, how many shisha (Ajami and/or Mouassal) do you smoke per week? (Answer by zero if you are a non-smoker) (open-ended question)
4. On average, how many pipes do you smoke per week? (Answer by zero if you are a non-smoker) (open-ended question)

**Part Three: Eye Cosmetics**

1. How often do you use inner eyeliner?

- Daily
- Once or twice per week
- Once or twice per month
- Don’t use/NA

1. How often do you use external eyeliner?

- Daily
- Once or twice per week
- Once or twice per month
- Don’t use/NA

1. How often do you use false eyelashes?

- Daily
- Once or twice per week
- Once or twice per month
- Don’t use/NA

1. How often do you use eye shadow?

- Daily
- Once or twice per week
- Once or twice per month
- Don’t use/NA

1. How often do you use mascara?

- Daily
- Once or twice per week
- Once or twice per month
- Don’t use/NA

1. How often do you use contact lenses?

- Daily
- Once or twice per week
- Once or twice per month
- Don’t use/NA

1. Duration since the start of using cosmetics:

- <6 months
- 6-12 months
- >12 months
- Don’t use/NA

1. Duration (in hours/day) of eye cosmetic use?

- < 6 hours
- Between 6 and 12 hours
- > 12 hours
- I don’t use eye cosmetics/NA

1. Which cleaning material is used to remove eye cosmetics?

- Water only
- Water with soap
- Cleansing cream
- I don’t use eye cosmetics/NA

1. Use of a cleanser to remove cosmetics:

- Rarely
- Sometimes
- Always
- I don’t use eye cosmetics/NA

1. Sleep routine:

- Sleep with eye cosmetics
- Sleep with lenses
- Sleep with both lenses and cosmetics
- Remove lenses or any cosmetic before sleeping
