# Supplementary material for: Incidence of dry eye symptoms and behavioural-cultural risk factors among university students population in Jordan
Source: PLoS One. 2025 Aug 7;20(8):e0328235. doi: 10.1371/journal.pone.0328235 (PMC12331033; doi:10.1371/journal.pone.0328235)
Supplement: Attachment — Submitted filename: Response to Reviewers.docx [file pone.0328235.s004.docx]

**Response to Reviewers**

We would like to sincerely thank the reviewers for their thorough evaluation of our manuscript and for providing valuable and insightful comments. We appreciate the time and effort invested in reviewing our work and recognize that the suggestions offered have significantly contributed to improving the clarity, rigor, and overall quality of the manuscript.

In the following document, we have addressed each comment carefully and made the necessary revisions to strengthen the study.

We hope that the changes meet the reviewers’ expectations and enhance the impact of our research.

**Reviewer #1:**

This study assessed the prevalence of symptomatic dry eye disease (DED) among university students in Jordan and its association with behavioral and cultural risk factors. Defined by an OSDI score ≥ 13, DED prevalence was 74.2%. They concluded that symptomatic DED is highly prevalent among university students in Jordan and is significantly associated with factors such as age, gender, contact lens use, cosmetic application, and tobacco consumption. The manuscript contains important findings; however, it needs to undergo revision to enhance its significance.

Abstract:
* The research objective needs to be more precise. You stated:

"To estimate the prevalence of symptomatic dry eye disease (DED) among university students in Jordan and examine the relationship between behavioral and cultural risk factors and the severity of DED."

However, you did not assess the prevalence of dry eye disease, as you did not diagnose DED according to standard criteria (OSDI symptom questionnaire≥ 13 plus at least one positive clinical test—NITBUT, osmolarity, or staining), as recommended by TFOS. Instead, you assessed the prevalence of dry eye symptoms, not the prevalence of individuals with dry eye disease who exhibit symptoms.

We acknowledge the distinction that the reviewer has highlighted between symptomatic dry eye and clinically diagnosed dry eye disease as defined by the TFOS DEWS II criteria. It is correct that our study utilized the OSDI questionnaire alone without accompanying clinical tests such as NITBUT, osmolarity, or ocular surface staining. Therefore, our findings pertain specifically to the prevalence of dry eye symptoms rather than confirmed dry eye disease. This is stated clearly in the manuscript title /aims*: “*Prevalence of Symptomatic Dry Eye Disease and Behavioural-Cultural Risk Factors among University Students population in Jordan”

In response to the reviewer’s comment, we will revise the objective in the manuscript to more accurately reflect this, as follows:
"To estimate the prevalence of *dry eye symptoms* among university students in Jordan and examine the relationship between behavioral and cultural risk factors and symptom severity. Additionally, the limitation of relying solely on symptoms without clinical confirmation of DED was acknowledged in the study limitations (lines 388-398)

*Rephrasing the objective and conclusions is necessary, especially considering that 61.3% of the participants had not been clinically diagnosed with DED.

Thank you for your insightful comment. We have revised both the objective and conclusions to clearly state that our results pertain to *dry eye symptoms* rather than clinically diagnosed *dry eye disease*. These revisions aim to ensure clarity and alignment with the data presented.

*The results section of the abstract should include the mean age of the participants, standard deviation, and age range.

*It is standard to include P-values and significant results within the results section of the abstract.

We sincerely thank the reviewer for this valuable comment. In response, we have revised the Results section of the abstract to include the mean age (21.87 years), standard deviation (3.824), and age range (18–45 years) of the study participants. Furthermore, we have incorporated the p-values corresponding to statistically significant findings to enhance the clarity and rigor of the results presentation. The revised abstract has been updated accordingly.

Introduction:
On page 3, lines 67-68, it is stated that the prevalence of dry eye disease in Jordan is 59%. How was dry eye diagnosed? Was it through a questionnaire alone, or were clinical signs also used?

Thank you for your comment and for pointing this out. The reported 59% prevalence of dry eye disease in Jordan, as cited on page 3 (lines 71-75), was based on symptoms **only** and assessed using a non-validated version of the OSDI questionnaire. No clinical signs or diagnostic tests were used in that previous study. We also **note** that this earlier report was co-authored by one of the current co-authors of this submission, which we acknowledge in the context of the literature cited.

To avoid any confusion, we will clarify this point in the manuscript lines 67-68 to ensure an accurate interpretation of the referenced prevalence figure.

Methods:
*The statistical analysis lacks a sample size calculation.

We acknowledge that a formal sample size calculation was not conducted prior to data collection, as the study adopted a convenience sampling method targeting university students across all Jordanian governorates. Our primary aim was to maximize participation by distributing the survey widely through social media platforms and university research departments, rather than limiting the sample to a predefined number.

Nonetheless, with a final sample of 788 participants, the study achieved a sufficiently large cohort to provide robust and generalizable findings for the target population. To strengthen the methodological transparency, we have now added a statement to the "Materials and Methods" section clarifying the sampling strategy and the absence of a priori sample size calculation.

*It is not stated whether a distribution test was performed on the data. Is the distribution normal or not?

ANOVA is known to be robust to violations of normality, particularly with large sample sizes. Given that the current sample size is 788, well above the commonly accepted thresholds (n > 30–40), the Central Limit Theorem ensures that the sampling distribution of the mean approximates normality. Therefore, the use of one-way ANOVA remains appropriate, and there was no need to switch to a nonparametric alternative [1].

Results:
*The mean age of the participants and standard deviation should be added. If the age distribution is not normal, the median and quartiles (Q1, Q3) should also be included.

We have revised the Results section to include the mean age and standard deviation of the participants.

The mean age of the participants was 21.87 years (SD = 3.824).

Discussion:
*It should be clarified that you assessed the prevalence of dry eye symptoms and not the prevalence of individuals with dry eye disease who are symptomatic (page 15, lines 308-310).

Thank you for your comment. We have revised the manuscript to clarify that our study assessed the prevalence of *symptomatic dry eye* rather than the prevalence of clinically diagnosed dry eye disease. The objective, results, discussion and conclusion sections have all been amended accordingly to accurately reflect this distinction. The text, has also been updated to ensure consistency with this clarification.

*It is recommended to discuss potential biases or methodological limitations that could account for the higher prevalence observed in this study compared to previous research.

We have expanded the discussion section to address potential biases and methodological limitations that may have contributed to the higher prevalence of dry eye symptoms observed in our study compared to previous research. Refer to Lines 392-401

**Reviewer #2:**

This represents an interesting study regarding the prevalence of dry eye symptoms.
What instructions were given to participants? The OSDI questionnaire contains questions referring to blurred vision that may be confused with uncorrected refractive error.

Why was the OSDI Cutoff Score of 13 used, rather than 20 as in previous study? (Bakkar et al Epidemiology of symptoms of dry eye disease (DED) in Jordan: Across-sectional non-clinical population-based study. Contact Lens and Anterior Eye [39 (2016) 197](callto:39%20(2016)%20197)–202). For better comparison with the general population in Jordan it would make sense to use the same cutoff score. Especially, as a score of 13 represents very mild symptoms.

Thank you for your thoughtful feedback. Participants were instructed to complete the OSDI questionnaire based on their subjective experience of dry eye symptoms during the previous two weeks, without consideration of any known refractive error or visual correction. We acknowledge that certain questions, particularly those referring to blurred vision, may have been influenced by uncorrected refractive error, which could introduce some response bias.

Regarding the cutoff score, yes, a previous report by Bakkar et al. used a cutoff of 20; however, we relied on more recent studies that use a cutoff value of 13 to define symptomatic dry eye, as this has become a widely accepted threshold in current literature. Using this cutoff improves consistency with more recent research and facilitates broader comparisons. This rationale has been clarified in the methods and discussion sections of the manuscript.

Line 68: the study only explored symptoms of DED, please adjust accordingly.

Thank you for your comment. We have revised the manuscript to clarify that our study assessed the prevalence of *symptomatic dry eye* rather than the prevalence of clinically diagnosed dry eye disease. The objective, results, discussion and conclusion sections have all been amended accordingly to accurately reflect this distinction. The text, has also been updated to ensure consistency with this clarification.

Line 169: please delete ‘although’ – Does this mean that 38.7 % of the participants have been previously diagnosed with DED? Please explain. If indeed, this large proportion of participants already have a DED diagnosis, a sub analysis of this group in view of risk factors may be warranted in view of the large sample size in this study.

We have removed the word "although" for clarity. To clarify, 38.7% of the participants self-reported a previous diagnosis of DED by an eye care professional. However, we acknowledge that this was based on self-reporting and not verified through medical records or standardized clinical criteria, which may introduce recall or reporting bias.

Given your helpful suggestion and the size of the sample, we agree that a sub-analysis of this subgroup could provide additional insights into potential differences in risk factor profiles. We have now included this as a consideration for future research in the discussion section.

Results:
In view of the very large standard deviations, it would be very interesting to include histograms similar to Figures 1-3 in your previous publication (Bakkar et al 2016).

We have included two histograms in the revised manuscript (Figure1 and Figure 2) to illustrate the distribution of electronic device usage hours and study hours across different DED severity groups. These visualizations provide a clearer understanding of the observed variability and support the interpretation of the relatively weak associations between these predictors and DED severity.

[1]	M. Blanca, R. Alarcón, J. Arnau, R. Bono, and R. Bendayan, ‘Non-normal data: Is ANOVA still a valid option?’, *Psicothema*, vol. 4, no. 29, pp. 552–557, Nov. 2017, doi: 10.7334/psicothema2016.383.
