# Supplementary material for: Incidence of dry eye symptoms and behavioural-cultural risk factors among university students population in Jordan
Source: PLoS One. 2025 Aug 7;20(8):e0328235. doi: 10.1371/journal.pone.0328235 (PMC12331033; doi:10.1371/journal.pone.0328235)
Supplement: Attachment — Submitted filename: Response_to_Reviewers_auresp_2.docx [file pone.0328235.s005.docx]

**Rebuttal letter**

Both reviewers feel that the revision addresses the comments. I still would like to see the authors refining their use of "symptomatic DED" which implies that the authors examined the presence of DED- which they did not, by replacing it with "dry eye symptoms". Similarly "severity of DED" should be replaced with "Severity of symptoms". All the terms “prevalence”, “symptomatic DED”, and “DED severity” were replaced by “incidence”, “Dye eye symptoms”, and “severity of symptoms”, respectively

Additional minor comments below as well:
Abstract
Your study only assessed incidence of symptoms and not of symptomatic dry eye disease- therefore, you must correctly refer to your study as assessing dry eye symptoms
Page 3 Line 88: the prevalence of dry eye symptoms, not symptomatic DED(corrected)
Page 4 Line 101: dry eye symptoms, not symptomatic DED (corrected)
Page 4: please calculate the power of a sample with 788 respondents precisely and not “provided a sufficiently large database”

A post hoc power analysis was conducted using **G*Power (version 3.1.9.7)** for the one-way ANOVA tests applied in the study. The total sample consisted of **788 university students,** and power calculations were performed for the two primary categorical independent variables used in the ANOVA models, which included variables with **4 groups** (smoking rate, smoking areas, and all the cosmetics variables) and **6 groups** (smoking type).

For the variable with **4 groups,** the analysis yielded a **non-centrality parameter (λ) = 49.25**, **numerator degrees of freedom (df) = 3, denominator df = 784**, a **critical F value = 2.616**, and a **statistical power (1 − β) = 0.999995.**

Similarly, for the variable with **6 groups,** the parameters were: **λ = 49.25, numerator df = 5, denominator df = 782, critical F value = 2.226,** and **statistical power = 0.999977.**

These results confirm that the achieved sample size was statistically robust and sufficiently powered to detect moderate effects across all ANOVA tests used in the study, thereby justifying the absence of an a priori sample size calculation.

This is now clearly addressed in the text in page 4, as follows:

*“To further verify the adequacy of the sample size, a* ***post hoc power analysis*** *was conducted using* ***G*Power (version 3.1.9.7)*** *for the* ***One-way ANOVA tests*** *employed in the study. Power estimates were calculated for variables with* ***four groups*** *(smoking rate, smoking areas, and all the cosmetics variables) and* ***6 groups*** *(smoking type), which represent the range of groupings used in the analysis. For the variable with four groups, the analysis yielded a* ***non-centrality parameter (λ) = 49.25, numerator df = 3, denominator df = 784, critical F = 2.616,*** *and a* ***power of 0.999995.*** *For the variable with six groups, the results were similarly robust:* ***λ = 49.25, numerator df = 5, denominator df = 782, critical F = 2.226,*** *and* ***power = 0.999977****. These results confirm that the sample size was statistically robust and sufficiently powered to detect moderate effects across the ANOVA models used in the study.”*

Page 8 Line 179: instead of “Regarding the participants’ field and year of study,” please rephrase: “Of the participants,” (corrected)
Pages 9-10 Line 201, 203, 207, 208, 215: it is not DED severity, it is dry eye symptom severity
Tables 2, 4, 5: severity of SYMPTOMS, not of DED- you do not know the severity of DED as you did not measure it at all (corrected)
Line 288, Line 293, Line 295, Line 304, Line 315, Line 326, Line 327m Line 329Line 331,Line 334, Line 341 (here it is stated as DED prevalence and severity, both were not examined), Line 342, Line 350,: It is NOT DED severity, it is Symptoms severity (corrected)
Line 348: used instead of utilized (corrected)
Page 19 Line 357: dry eye symptoms and not symptomatic DED (corrected)
Line 359: severity of symptoms, not symptomatic DED (corrected)
Line 378: dry eye symptoms (corrected)
Line 389: dry eye symptoms (corrected)
Line 423: dry eye symptom severity (corrected)
Line 431: studies instead of research (corrected)
Line 449: dry eye symptoms instead of symptomatic DED (corrected)
Line 451: the Jordanian population (corrected)
