# Supplementary material for: Incidence of dry eye symptoms and behavioural-cultural risk factors among university students population in Jordan
Source: PLoS One. 2025 Aug 7;20(8):e0328235. doi: 10.1371/journal.pone.0328235 (PMC12331033; doi:10.1371/journal.pone.0328235)
Supplement: Attachment — Submitted filename: Response to Reviewers (2).docx [file pone.0328235.s006.docx]

**Rebuttal letter**

The authors should re-read their manuscript and refer to symptoms of dry eye when they are referring to their study which is only based on OSDI. However, in the introduction and discussion - in instances where the authors quote previous studies which examined dry eye disease using clinically acceptable diagnostic criteria- they should not refer to dry eye symptoms. The way the paper was submitted appears as if the authors replaced every single instance of the words "dry eye" with "dry eye symptoms (DES)" including places such as the TFOS guidelines for diagnosing dry eye disease (and not just symptoms).
Further DES- is often used for digital eye strain and even dry eye syndrome. Please refrain from the abbreviation and write DE symptoms.
The authors are requested to please seriously review and read their paper before resubmitting a manuscript in the current state. All the suggested comments by the editor were considered in the revised manuscript.
